# The impact of body vigilance on help-seeking for cancer ‘alarm’ symptoms: a community-based survey

**DOI:** 10.1186/s12889-016-3846-7

**Published:** 2016-11-21

**Authors:** Kelly Winstanley, Cristina Renzi, Claire Friedemann Smith, Jane Wardle, Katriina L. Whitaker

**Affiliations:** 1 Health Behaviour Research Centre, Department of Epidemiology and Public Health, University College London, London, WC1E 6BT UK; 2 School of Health Sciences, University of Surrey, Guildford, Surrey GU2 7XH UK

**Keywords:** Body vigilance, Early diagnosis, Cancer, Help-seeking, Symptoms

## Abstract

**Background:**

The act of detecting bodily changes is a pre-requisite for subsequent responses to symptoms, such as seeking medical help. This is the first study to explore associations between self-reported body vigilance and help-seeking in a community sample currently experiencing cancer ‘alarm’ symptoms.

**Methods:**

Using a cross-sectional study design, a ‘health survey’ was mailed through primary care practices to 4913 UK adults (age ≥50 years, no cancer diagnosis), asking about symptom experiences and medical help-seeking over the previous three months. Body vigilance, cancer worry and current illness were assessed with a small number of self-report items derived from existing measures.

**Results:**

The response rate was 42% (*N* = 2042). Almost half the respondents (936/2042; 46%) experienced at least one cancer alarm symptom. Results from logistic regression analysis revealed that paying more attention to bodily changes was significantly associated with help-seeking for cancer symptoms (OR = 1.44; 1.06-1.97), after controlling for socio-demographics, current illness and cancer worry. Being more sensitive to bodily changes was not significantly associated with help-seeking.

**Conclusions:**

Respondents who paid attention to their bodily changes were more likely to seek help for their symptoms. Although the use of a cross-sectional study design and the limited assessment of key variables preclude any firm conclusions, encouraging people to be body vigilant may contribute towards earlier cancer diagnosis. More needs to be understood about the impact this might have on cancer-related anxiety.

## Background

The World Health Organisation highlights two major components of early cancer detection: education to promote recognition of warning signs; and screening [1]. As relatively few cancers are detected through screening [2], understanding how people recognise and interpret bodily changes is central to improving cancer outcomes [3, 4]. The Model of Pathways to Treatment (MPT) defines the patient interval as time from first experiencing a bodily change to first consultation with a healthcare professional, and is divided into appraisal and help-seeking components [3].

Although detecting relevant bodily changes is the first step towards recognising cancer symptoms, research has mainly focused on the help-seeking interval (time from perceiving a reason to contact a healthcare professional to first consultation) [3, 5–7]. However, delay associated with symptom appraisal (time from detecting bodily changes to perceiving a reason to discuss symptoms with healthcare professional) is considered a key factor influencing time to diagnosis [8], accounting for at least 60% of patients’ total delay [9, 10]. Qualitative evidence using MPT as a framework suggests that key factors prolonging the appraisal interval include patient factors (e.g. misinterpretation, attributing symptoms to benign causes, comorbidities) and disease factors (e.g. disease site) [11–13].

Symptom appraisal models are helpful in further unpacking patient response processes in the appraisal interval stage [14]. For example, attention appears to be critically important in the detection phase because self-focused attention is needed to detect bodily changes [14, 15]. To date, body vigilance, defined as conscious attention focused on bodily sensations [16], has mainly been researched in terms of its association with anxiety-related constructs, such as anxiety sensitivity [17] and panic disorder [16]. However, it may also be an important construct to study in the context of cancer symptom identification and presentation.

Studies in non-clinical samples and those with anxiety disorders have suggested that heightened body vigilance can enhance medical help-seeking [18]. Yet to our knowledge, no studies have explored associations between body vigilance and help-seeking among those experiencing possible cancer symptoms. One study among asymptomatic Dutch adults found women and the more educated were more likely to anticipate paying attention to cancer symptoms [19]. However, attention to cancer symptoms was not directly assessed in this research and there was no assessment of help-seeking.

The Body Vigilance Scale [16], which is a widely used measure of body vigilance and has been validated in non-clinical samples, includes two items that may be relevant in the context of earlier diagnosis. One of these items is related to whether individuals pay close attention to their bodily sensations, whereas the other is related to whether individuals are sensitive to bodily sensations. The aim of the current study was to examine the association between these two elements of self-reported body vigilance and help-seeking among adults reporting cancer ‘alarm’ symptoms.

## Methods

### Study design and recruitment of participants

A detailed description of the study methods has been given previously [20, 21]. Briefly, in October 2013, 4931 questionnaires were sent to patients registered at four general practices across London, the South East and the North West of England. Participants were men and women aged ≥50 years, with no current cancer diagnosis. Non-responders received a reminder letter and questionnaire pack after 2 weeks.

### The questionnaire

#### Symptom experience and help seeking

The questionnaire was presented as a general health survey to avoid alerting people to the cancer context. Respondents were asked whether they had experienced one or more of 14 cancer ‘alarm’ symptom in the past three months (Table 1). Ten were taken from the Cancer Awareness Measure [22], with an additional four from the Be Clear on Cancer Campaigns [23]. For each symptom reported in the last three months, respondents were asked whether they had sought help from a General Practitioner (GP) (‘yes’ or ‘no’).

**Table 1:** Cancer ‘alarm’ symptoms

Persistent cough or hoarseness	
Unexplained lump	
Unexplained weight loss	
Change in the appearance of a mole or a new mole	
Persistent change in bowel habits	
Persistent change in bladder habits	
Abdominal bloating (i.e. bloating of the tummy or belly)	
Unexplained pain	
Difficulty swallowing	
Blood in urine	
Rectal bleeding (i.e. bleeding from the back passage or blood in the bowel motions)	
Other unexplained bleeding	
Any breast changes	
A sore that does not heal	

#### Body vigilance

Two items assessing body vigilance ‘I am very sensitive to changes in my body’ (BV-sensitivity) and ‘I pay close attention to changes in my body’ (BV-attention) were selected and adapted from the Body Vigilance Scale [16]. The items were considered independently because of their semantic and conceptual differences; one assessed perceived sensitivity to bodily changes (a more reactive, passive response) and the other assessed degree of attentional focus (suggestive of a more pre-emptive form of vigilance). Responses were on a five-point Likert Scale from ‘strongly disagree’ to ‘strongly agree’.

#### Cancer worry

A measure of cancer worry was included based on evidence that it is associated with body vigilance, and may be a potential confounder in the relationship between body vigilance and help-seeking. Worry about cancer was measured with a question taken from Berrenberg’s cancer attitude inventory [24], ‘On a day-to-day basis, how much do you worry about cancer’. Responses were on a five-point Likert scale (from ‘not at all’ to ‘a lot’). The question was embedded among items on worry about two non-cancer diseases to mask the cancer context.

#### Socio-demographic characteristics

Respondents were asked for their age (categorised for analysis as 50–59; 60–79; 80+), ethnicity (white versus non-white ethnic background), highest level of education (degree or higher versus below degree), marital status (married/cohabiting versus not married/cohabiting) and employment status (working versus not working). They were also asked whether they had a current diagnosis of cancer (yes/no) or if they had any current illness that interfered with their daily life (yes/no).

### Statistical analysis

Data were analysed using SPSS 22.0. Spearman’s correlations were calculated for the two body vigilance items. As body vigilance is considered an anxiety-related construct [16], correlations between each of the body vigilance items and cancer worry were also examined.

Cancer worry was categorised into *Low* (not at all, a little or moderately) and *High* (quite a bit, a lot). Both body vigilance items were dichotomised as *Endorsed* (‘slightly or strongly agree’) or *Not Endorsed* (‘neither agree nor disagree’, ‘slightly or strongly disagree’). Categorisation of variables was based on our previous research to allow results to be comparable [25].

Univariable analysis, namely logistic regression, was initially used to explore associations between socio-demographic, psychological variables and help-seeking. Variables that were associated with help-seeking in univariable analyses at *p* < 0.25 [26] were entered into the multivariable logistic regression models. Two multivariable models were used to explore whether the two elements of body vigilance were independently associated with help-seeking.

## Results

### Response

A total of 2042 people returned completed questionnaires (response rate = 42%). Respondents were 54% female with an average age of 65 (range: 50–100 years). Most (94%) were white and 68% were married or co-habiting, 37% were educated to university level, and 41% were employed. These demographics are comparable to the profile of over 50 year olds in England [25], although non-white ethnic groups were under-represented. Symptomatic respondents were more likely to be female [X^2^ (1) = 16.81, *p* < .01)], single [X^2^ (1) = 17.93, *p* < .01)], have a current illness [X^2^ (1) = 66.27, *p* < .01)], report high cancer worry ([X^2^ (1) = 20.66, *p* < .01)] and be sensitive to bodily changes [X^2^ (1) = 5.43, *p* < .05)], compared to asymptomatic respondents (Table 2).

**Table 2:** Demographic characteristics among symptomatic and asymptomatic respondents

		Symptomatic (*N* = 917) n (%)	Asymptomatic (*N* = 1106) n (%)
Sex	Male	374 (40.8)	551 (49.8)
	Female	534 (58.2)	543 (49.1)
Age	50–59	318 (34.7)	348 (31.5)
	60–79	492 (53.7)	653 (59.0)
	80+	77 (8.4)	74 (6.7)
Marital Status	Single/Not co-habiting	336 (36.6)	307 (27.8)
	Married/Co-habiting	573 (62.5)	786 (71.1)
Education	Below degree level	554 (60.4)	697 (63.0)
	Degree or higher	341 (37.2)	389 (35.2)
Employment	Not working	538 (58.7)	640 (57.9)
	Working	368 (40.1)	451 (40.8)
Ethnicity	Non-white	53 (5.8)	54 (4.9)
	White	858 (93.6)	1046 (94.6)
Cancer worry	Low cancer worry	739 (80.6)	984 (93.4)
	High cancer worry	108 (11.8)	70 (6.6)
Current illness	No	465 (50.7)	756 (68.4)
	Yes	422 (46.0)	319 (28.8)
BV-sensitivity	No	470 (51.3)	622 (56.2)
	Yes	425 (46.3)	455 (41.1)
BV-attention	No	438 (47.8)	539 (48.7)
	Yes	468 (51.0)	549 (49.6)

Column totals may vary due to missing data (ranging from <1% (*n* = 6 for ethnicity to 3% (*n* = 30) for age and current illness). *BV-sensitivity* ‘I am very sensitive to changes in my body’, *BV-attention* ‘I pay close attention to changes in my body’

Non-responder analysis showed that the probability of not responding was greater for men (39.4%) than women (44.3%) [X^2^ (1) = 11.70, *p* < .01)], and for 50–59 year olds (34.2%) than 60–69 year olds (49.4%) or those 70 and over (45.3%) [X^2^ (2) = 92.48, *p* < .001].

Almost half the sample (46%; 936/2042) reported experiencing at least one cancer alarm symptom over the previous three months. Of these, 18 were excluded as they reported a diagnosis of cancer and 1 was excluded because they had missing data for the ‘cancer diagnosis’ variable, resulting in a final sample for analysis of *n* = 917. The most commonly reported symptoms were persistent cough or hoarseness (23%), persistent change in bowel habits (18%), and persistent change in bladder habits (17%). The least commonly reported symptoms were blood in urine (5%), difficulty swallowing (5%), other unexplained bleeding (3%) and breast changes (3%).

Most of the symptomatic patients (*N* = 859) reported whether they had sought help for their symptom/s during the last 3 months, with 63% (542/859) having visited a GP. Spearman’s correlations revealed a large and significant correlation between the two body vigilance items (*r* = .55, *p* < .001). There were also small but significant correlations between cancer worry and BV-attention (*r* = 0.12, *p* < .01), and BV-sensitivity (*r* = 0.15, *p* < .001).

### Association between body vigilance and help-seeking

Logistic regression models are presented in Table 3. BV-attention was associated with a significantly higher likelihood of seeking help for at least one cancer alarm symptom in multivariable analyses (OR = 1.44, 1.06-1.97). BV-sensitivity was not associated with help-seeking in multivariable analyses (OR = 1.02, 0.75-1.40).

**Table 3:** Multivariable logistic regression models for the association between each of the body vigilance items (BV-sensitivity and BV- attention) and help-seeking, controlling for potential confounders

		Sought help for one or more symptom			
		N (%) sought help	Univariable association between confounder and help-seeking (*p* value)	Model including BV-sensitivity Adjusted OR^a^ (95% CI)	Model including BV-attention Adjusted OR^a^ (95% CI)
Sex	Male	214 (61.3)	0.40	--	--
	Female	322 (64.1)			
Age	50–59	183 (60.0)	0.30	--	--
	60–79	304 (65.5)			
	80+	40 (62.5)			
Marital Status	Single/Not co-habiting	196 (63.0)	0.90	--	--
	Married/Co-habiting	344 (63.5)			
Education	No higher education	334 (65.0)	0.11	1.00	1.00
	Degree or higher	196 (59.6)		0.85 (0.62–1.16)	0.83 (0.61–1.14)
Employment	Working	196 (55.7)	0.00	1.00	1.00
	Not Working	342 (69.0)		**1.53 (1.11–2.09)**	**1.55 (1.13–2.13)**
Ethnicity	Non-white	38 (73.1)	0.13	1.00	1.00
	White	510 (62.5)		0.60 (0.31–1.18)	0.58 (0.29–1.14)
Cancer worry	Low worry	429(61.5)	0.13	1.00	1.00
	High worry	70 (69.3)		1.45 (0.89–2.35)	1.37 (0.84–2.23)
Current illness	No	237 (54.4)	0.00	1.00	1.00
	Yes	286 (72.4)		**2.11 (1.53–2.90)**	**2.08 (1.51–2.86)**
BV-sensitivity	Not endorsed	229 (62.1)		1.00	--
	Endorsed	261 (64.3)		1.02 (0.75–1.40)	
BV-attention	Not endorsed	231 (57.6)		--	1.00
	Endorsed	303 (67.6)			**1.44 (1.06–1.97)**

^a^Adjusted for education, employment, ethnicity, cancer worry and current illness. Highlighted figures are statistically significant (*p* < 0.05). *OR* odds ratio, *CI* confidence interval, *BV-sensitivity* ‘I am very sensitive to changes in my body’, *BV-attention* ‘I pay close attention to changes in my body’

In both regression models, unemployed/retired people were significantly more likely to have sought help for at least one symptom (*p* < .01) and reporting a current illness was also associated with being more likely to have sought help (*p* < .001).

## Discussion

This is the first large-scale survey to explore associations between body vigilance and help-seeking for people reporting cancer ‘alarm’ symptoms. The key finding was that people who paid more attention to their body were more likely to have sought help from their doctor. Despite the association between cancer worry and body vigilance, and the high positive correlation between ‘paying attention’ and ‘being sensitive’ to bodily changes, only ‘paying attention’ was positively associated with help-seeking. Being unemployed/retired and/or reporting a current illness were also found to be independently associated with increased likelihood of help-seeking in multivariable analysis.

This is the first time the relationship between body vigilance and help-seeking has been demonstrated in the cancer context, and supports theoretical models of symptom appraisal emphasising the importance of attentional process in help-seeking for cancer ‘alarm’ symptoms [14]. Although work on the relationship between body vigilance and cancer diagnosis is scarce, a recent prospective study in Norway found health anxiety (defined as persistent preoccupation with developing a serious medical condition) was associated with increased likelihood of a cancer diagnosis in men, but not women [27]. It was suggested that men with high levels of health anxiety are more likely to detect a malignant tumour than men with lower levels of anxiety, which may aid early cancer detection. However the mechanism by which anxiety led to higher incidence of cancer diagnosis remains unclear.

Caution was also noted because health anxiety may lead to overdiagnosis and overtreatment [27]. It is thus important to distinguish between ‘paying attention’ (getting to know your body and being aware of changes) and active self-checking. For example, for breast cancer, women who regularly check their breasts are almost twice as likely to have a biopsy of a benign lump but are no less likely to die from cancer, and therefore active self-checking is often not recommended [28]. However, promoting body vigilance in developing countries where, for example, women present with late stage disease of breast cancer may be beneficial [29, 30].

There was no significant association between cancer worry and help-seeking, and body vigilance was independently associated with help-seeking. One potential explanation is the mixed role of emotion in help-seeking [31], where cancer worry can act both as a barrier and facilitator to contacting a health care professional [32].

In line with existing research, unemployed/retired people were more likely to seek help for their symptoms [25]. This may be explained by unemployed/retired people having less competing stimuli for their attention and therefore more cognitive resources available for noticing and responding to internal bodily changes [33]. Respondents reporting current illnesses were also more likely to have sought help for their symptom, and this was the strongest predictor of help-seeking in the current study in multivariable analysis. This supports previous research where people described mentioning a worrying symptom when consulting about something else [34].

### Limitations

Despite being broadly representative of over 50 year olds in the UK [25], non-white ethnic groups were under-represented in this study, and targeted work on body vigilance in different communities may be beneficial. Our response rate was 42%, which is higher than other primary care based surveys [35]. However, the finding that men and younger people were less likely to respond limits the generalisability of these findings.

As recognised previously [20], the bias associated with questionnaire return cannot be estimated - experience of symptoms could both encourage and discourage it. For example, people who do not respond to surveys may be more avoidant generally, and less likely to attend to bodily changes or seek help. The prevalence of hypochondriasis in the current population is also unknown, and as this is associated with elevated body vigilance [18], may also influence questionnaire return. However, in population-based studies prevalence rates of hypochondriasis are estimated to range between 0.2 and 4.5% [36] so this is unlikely to have had a significant impact on the findings of the present study.

It should also be noted that the use of a cross-sectional study design limits any inferences concerning the directions of the observed associations. For example, certain forms of help-seeking may be conducive to greater body vigilance or associations may exist in both directions.

Our findings are based on self-reported body vigilance using items developed outside the cancer field, and single items were used to assess cancer worry, help seeking, and each component of body vigilance, which could raise questions about their reliability and validity. It might be interesting to qualify what people mean when reporting ‘attention’ to bodily changes to clarify conceptual differences between sensitivity to bodily changes compared to being attentive. This information could advance our understanding in terms of whether and how people distinguish between these two types of body vigilance. A number of cancer ‘alarm’ symptoms were pooled in the present analyses but future research could explore the influence of body vigilance at the symptom level.

There could be some concern over whether encouraging body vigilance might lead people to become overly anxious about bodily changes, particularly in light of established associations between anxiety and body vigilance [18] . Similarly, some may worry that encouraging people attend to unusual changes may direct a large number of people to primary care, possibly overloading the healthcare system with the ‘worried well’ [37]. In most cases, ‘alarm’ symptoms will not be indicative of cancer [38], and body vigilance interventions should be designed so they do not provoke undue anxiety or encourage unnecessary help-seeking. To further understand the implications of these findings, future longitudinal studies could explore the impact of body vigilance on help-seeking behaviour.

## Conclusion

People who paid attention to bodily changes were more likely to seek help for cancer ‘alarm’ symptoms. Efforts to encourage body vigilance without causing excessive anxiety could be useful when designing early detection interventions. In turn, this could result in timelier help-seeking and may contribute towards earlier cancer diagnosis.

## Abbreviations

BV-attention: “I pay close attention to changes in my body”
BV-sensitivity: “I am very sensitive to changes in my body”
CI: Confidence interval
GP: General Practitioner
MPT: Model of Pathways to Treatment
OR: Odds ratio
